# Sex‐specific differences in patients with nonmelanoma skin cancer of the pinna

**DOI:** 10.1002/hed.26237

**Published:** 2020-05-05

**Authors:** Ulrich Moser, Alexandros Andrianakis, Prisca Pondorfer, Axel Wolf, Matthias Graupp, Thomas Weiland, Clemens Holzmeister, Dominik Wild, Dietmar Thurnher

**Affiliations:** ^1^ Department of Otorhinolaryngology, Head and Neck Surgery Medical University of Graz Graz Austria; ^2^ Department of Otorhinolaryngology Krankenhaus der Barmherzigen Schwestern Ried Ried im Innkreis Austria

**Keywords:** basal cell carcinoma, sex, nonmelanoma skin cancer, pinna, squamous cell carcinoma

## Abstract

**Background:**

Generally, it is known that men are affected more frequently by nonmelanoma skin cancer (NMSC) than women. The aim of our study was to investigate the effect of sex on the characteristics of NMSCs of the pinna at the population that our center serves and to compare it with the international data.

**Methods:**

We analyzed retrospectively the data of 225 patients with NMSC of the pinna. Sex‐specific differences were investigated for basal cell carcinoma (BCC) and cutaneous squamous cell carcinoma (cSCC) subgroups.

**Results:**

The ratio of BCC to cSCC was determined in male patients at 1:1.3, in contrast in females it was identified at 4:1 (*P* = .001).

**Conclusion:**

In our study, a new aspect of the sex‐dependent distribution of cSCC and BCC of the pinna was demonstrated. Women are affected four times more frequently by BCC than by cSCC, whereas in men this ratio is approximately equal.

## 1 INTRODUCTION

Nonmelanoma skin cancer (NMSC) accounts for the most frequent entity of cancer worldwide.^1^ In general, basal cell carcinomas (BCCs) represent 75% to 80% of all cases of NMSC while cutaneous squamous cell carcinomas (cSCCs) are responsible for most of the remaining.^2^, ^3^ Other rare entities, which represents just under 1% of all NMSCs,^4^ include the basosquamous carcinoma, which is thought to be an aggressive subtype of BCC with an increased risk of recurrence and metastases^5^ or the rare but highly malignant Merkel cell carcinoma.^6^ Approximately 90% of NMSC are located in the head and neck region, of which 6% to 10% appear at the pinna.^7^, ^8^ In women, skin cancer is located more frequently in the midface while men are more often affected on the scalp and pinna.^9^ Skin cancer is rarely seen in the young population, but due to the accumulative effect of the risk factors during a lifetime the incidence increases with age.^10^, ^11^ In different countries, the incidence rate of NMSC varies significantly. In Germany, the incidence rate of BCC is appointed at 113:100.000 for men and at 102:100.000 for women, for cSCC it is indicated at 30:100.000 for men and at 16:100.000 for women.^9^ Overall the incidence of BCC and cSCC is still increasing.^12^, ^13^

The risk factors for NMSC can be divided in exogenic and endogenic factors. The most important exogenic factor is the ultraviolet radiation which causes molecular transformations in skin cells due to DNA‐initiated mutations.^14^ Additionally the radiation has effects on the inflammatory response of the skin and the subsequent migration of cells of the immune system which contribute to the development of skin cancer.^15^ An immunosuppressive therapy increases the risk of skin cancer, especially for SCC.^16^ Genetic predisposition like Fitzpatrick skin type 1, xeroderma pigmentosum, or variations in the Melanocortinreceptor 1 (MC1R) gene are also major risk factors for the development of BCC^17^, ^18^ and cSCC.^12^, ^19^ In a recent study, the possible role of oncogenic viruses, especially human papilloma viruses, in the origin of NMSC is postulated.^20^ cSCCs usually develop from UV‐induced predamaged skin lesions like actinic keratosis or Bowen's disease (carcinoma in situ), but can also arise de novo.^11^ For actinic keratosis, the risk of progression to cSCC is 0.6% at the first year and 2.6% at the fourth year.^21^ About 65% of all cSCCs emerge from precursor lesions.^22^ For BCCs, there are no known precursor lesions.^23^

The therapy of choice for NMSC is surgical excision with clear resection margins.^24^ Especially in the facial area, this excision is impeded by the task to achieve an appropriate cancer‐free safety distance as well as a satisfying aesthetic outcome. Specific facial aesthetic units may also be used for prognostic evaluation and follow‐up in NMSC, as reported for melanoma.^25^

BCCs are often classified as semimalignant because of their locally invasive growth but a very low rate of distant or regional metastases (<0.1%).^26^ Generally, the rate of locoregional metastasis of cSCCs lies between 0.5% and 2%. If located on the pinna, the risk increases up to 16%.^27^, ^28^ Hence, cSCCs of the external ear are associated with a higher risk of recurrence and disease progression, which leads to a poorer prognosis compared to cSCCs located at other body parts.^29^, ^30^

In general, men are more frequently affected by NMSC than women.^31^ The aim of our study was to investigate the effect of sex on the characteristics of NMSC at the pinna that our center serves and to compare it with the international data.

## 2 MATERIALS AND METHODS

The records of all patients with NMSC of the pinna treated at the Department of Otorhinolaryngology – Head and Neck Surgery of the Medical University of Graz from 2005 to 2015 were reviewed retrospectively. Inclusion criteria were histologic proof of cSCC or BCC and age of at least 18 years. Any other kind of skin cancer was excluded. The follow‐up period started at the time of diagnosis of malignant skin cancer and study endpoints were recurrence, death, or the end of data collection December 2015. To ensure an at least one‐year long surveillance, new patients were included not later than December 2014. For cSCC, the staging process included computer tomography or magnetic resonance tomography of head and neck, chest X‐ray, and abdominal ultrasonography. The TNM staging was used corresponding to American Joint Committee on Cancer of the year 2002—Union Internationale Contre le Cancer (UICC) 6th Edition or the year 2010—UICC 7th Edition, as appropriate.

The two histopathological groups cSCC and BCC were examined to look for sex differences in relation to age at presentation, tumor localization, TNM classification, grade of differentiation, recurrence, metastatic rate, recurrence free survival time, cartilage or bone infiltration, and treatment methods. Statistical analysis was operated with SPSS software, version 23.0 (SPSS, Chicago, Illinois). The data are presented as medians with ranges or means with SDs, as appropriate. Comparisons of continuous or categorical variables were performed with Student's *t* test or Mann‐Whitney *U* test and Chi‐squared or Fisher exact test, as appropriate. Kaplan‐Meier analysis was used to estimate recurrence free survival time and significances were identified with log‐rank test. Statistical significance was set at <.05 (two‐sided). The study was approved by the ethics committee of the Medical University of Graz.

## 3 RESULTS

**FIGURE 1:**
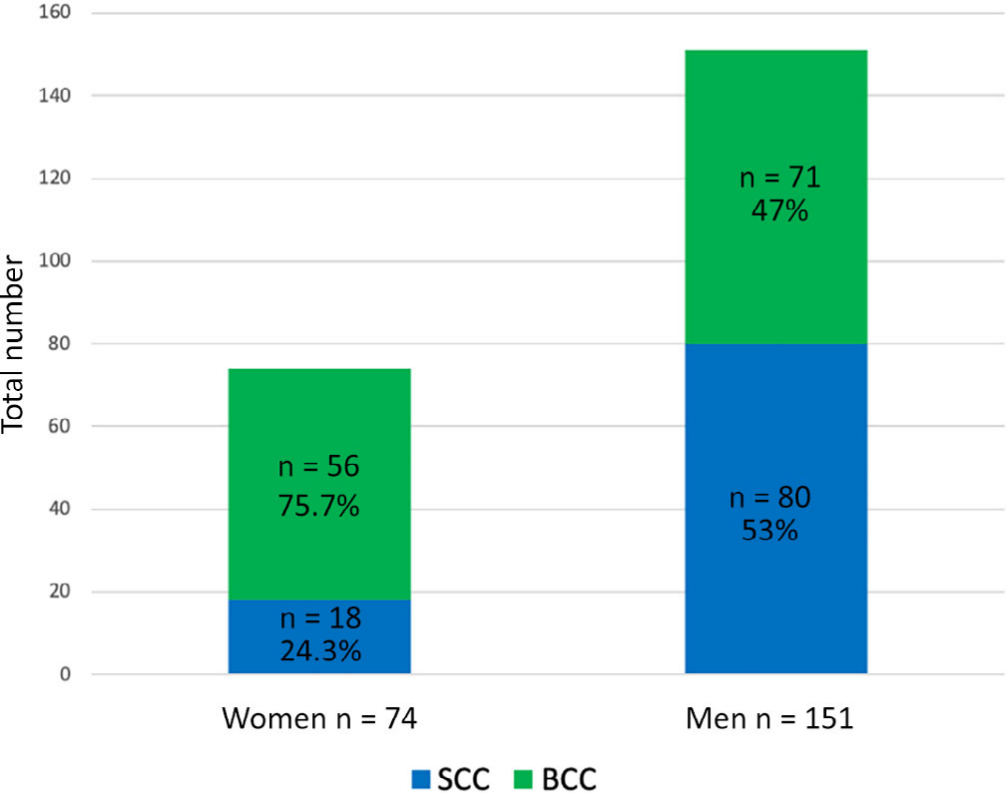
Sex‐specific incidences of squamous cell carcinoma and basal cell carcinoma [Color figure can be viewed at wileyonlinelibrary.com]

In total, 225 patients with NMSC of the pinna were included in our survey, to be specific 151 males (67.1%) and 74 females (32.9%). Regarding the histologic diagnosis, BCC was the most frequent kind (127 individuals [56.4%]) followed by cSCC (98 [43.6%]). We found that women were rarely diagnosed with cSCC (18 [23.7%]) and much more frequent with BCC (56 [73.7%]). On the contrary, the occurrence in the male study population was nearly balanced for cSCC (80 [53%]) and BCC (71 [47%]). Therefore, the distribution of BCC to cSCC in the female subgroup was approximately 4:1, while in the male group the ratio of BCC to cSCC was approximately 1.1:1 (*P* ≤ .001). Figure 1 outlines sex‐specific incidences of cSCC and BCC.

Generally, the age at diagnosis of cSCC (75.8 ± 10.3 years) was significantly higher than for BCC (72.7 ± 11.3 years) (*P* = .03). Women with cSCC of the pinna were significantly older than men, to be specific women had a median age of 81.2 ± 7.3 years whereas men were 74.6 ± 10.6 years old (*P* = .003). Also, in cases of BCC, the age at diagnosis was higher in women compared to men, to be specific 73.6 ± 12.0 years in women and 71.8 ± 10.7 years in men. Table 1 shows characteristics of male and female patients.

**TABLE 1:** Characteristics of male and female patients divided in SCC and BCC

Characteristics	Female	Male	Significance
Number SCC	18	80	*P* = .001
Age at diagnose SCC	81.2 ± 7.3	74.6 ± 10.6	*P* = .003
Recurrence rate SCC	16.7%	27.5%	*P* = .26
Number BCC	56	71	*P* = .001
Age at diagnose BCC	73.6 ± 12.0	71.8 ± 10.7	*P* = .34
Recurrence rate BCC	25.0%	12.7%	*P* = .06

Abbreviations: BCC, basal cell carcinoma; SCC, squamous cell carcinoma.

**FIGURE 2:**
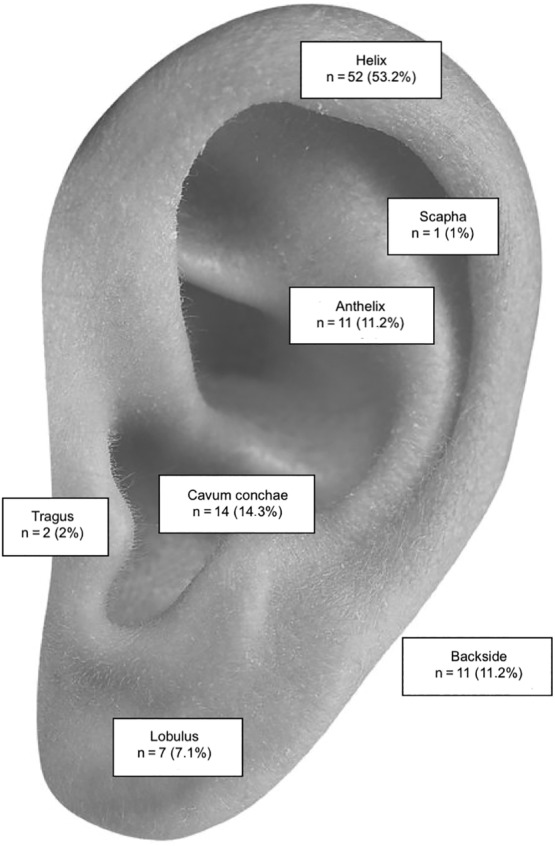
Location of SCCs. SCC, squamous cell carcinoma

**FIGURE 3:**
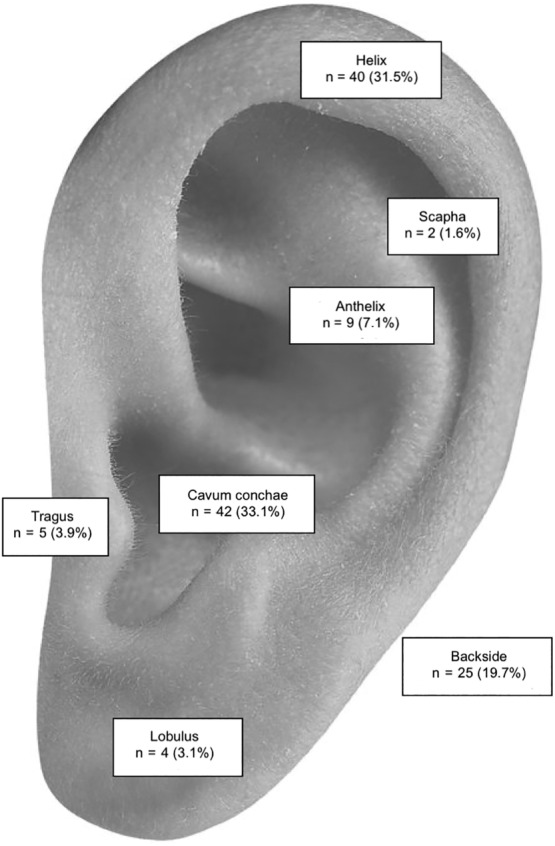
Location of BCCs. BCC, basal cell carcinoma

In women, the most common tumor site of cSCCs was the helix (66.7%), while BCCs were distributed almost equal between the helix (37.5%) and the cavum conchae (35.7%). In men, the helix (50%) was the most frequent location of cSCCs and the most common sites for BCCs were the cavum conchae (31%) and the helix (26.8%). When the distribution of the tumor site was compared dependent on sex a tendency toward higher rates of skin cancer in the backside of the pinna could be detected in men but no statistically significant discrepancy was visible. Figures 2 and 3 show detailed information of tumor locations of cSCCs and BCCs.

The infiltration of cartilage was significantly higher in cSCC than in BCC, in women in 26.7% of cSCC and in 6% of BCC (*P* = .04) and in men in 21.2% of cSCC and just in 8.3% of BCC (*P* = .05). The TNM classification and the grade of differentiation showed no significant differences referring to sex. The rate of nodal metastases was almost equal at 5.9% for women and at 5.2% for men. Table 2 shows the various characteristics in which sex‐specific differences were observed. The treatment of choice for the majority of primary cases was a surgical resection of the carcinoma in 219 patients (97.3%). In four cases (1.8%), a postoperative radiotherapy/chemotherapy was added. Due to the inoperability of the tumor or the patients unsuitability for a general anesthesia, a primary radiation therapy was performed in two further cases (0.9%). The local recurrence rate for men and women together was determined for cSCC at 25.5% and for BCC at 18.1%. For cSCC, the recurrence rate was found to be 27.5% for men and 16.7% for women. In cases of BCC, the rate of recurrence was 12.7% for men and 25% for women. These observed differences did not quite reach statistical significance (*P* = .06). In cases of recurrent disease, the treatment of choice was surgery in 29 patients (58%), surgery with postoperative radiotherapy/chemotherapy in 17 cases (34%), and a definitive radiotherapy/chemotherapy in 4 cases (8%).

**TABLE 2:** Statistical differences in sex‐specific characteristics

	Sex
Ratio SCC/BCC	*P* = .001
Age SCC	*P* = .003
Recurrence SCC	n.s.
Age BCC	n.s.
Recurrence BCC	*P* = .06
Follow‐up	n.s.
Therapy	n.s.
‐classification	n.s.
Tumor location	n.s
Grade of differentiation	n.s.
Cartilagineus infiltration	n.s.
Skullbase infiltration	n.s.
Parotic infiltration	n.s.
Nodal metastatic rate	n.s.

Abbreviations: BCC, basal cell carcinoma; n.s., no significance; SCC, squamous cell carcinoma.

**FIGURE 4:**
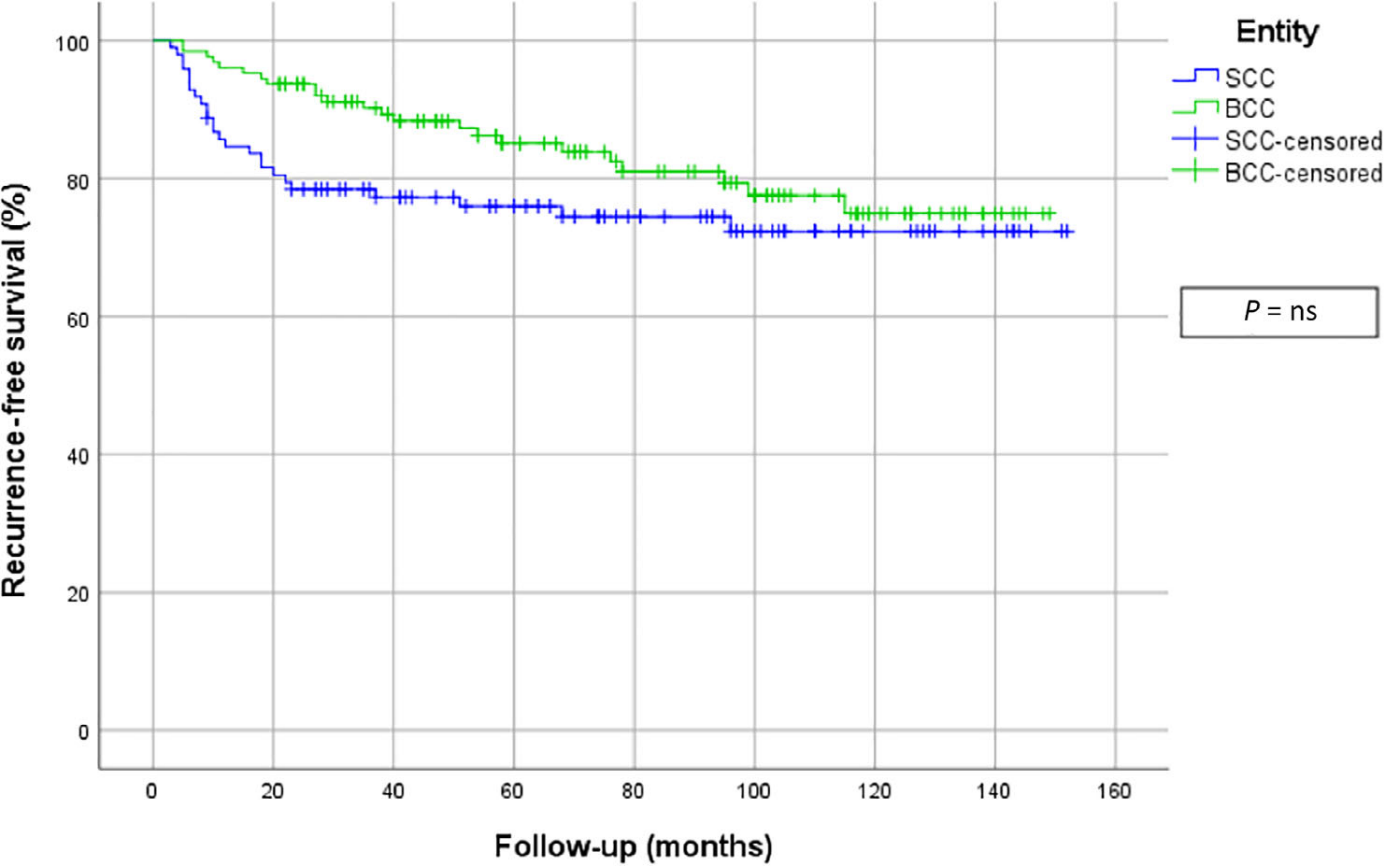
Recurrence‐free survival curves of all patients [Color figure can be viewed at wileyonlinelibrary.com]

**FIGURE 5:**
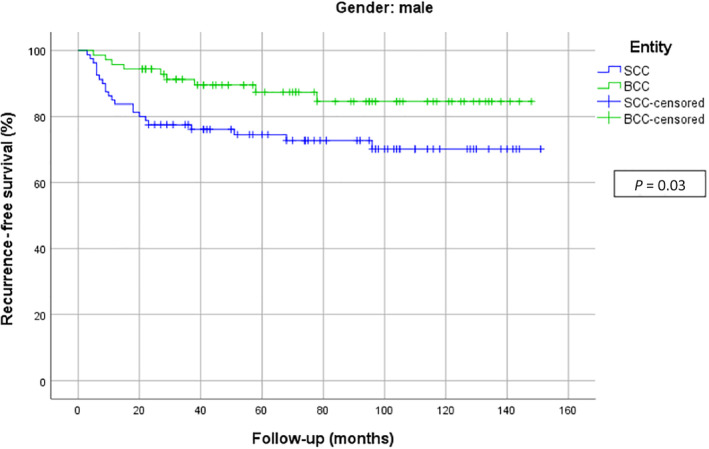
Recurrence‐free survival curves for men [Color figure can be viewed at wileyonlinelibrary.com]

**FIGURE 6:**
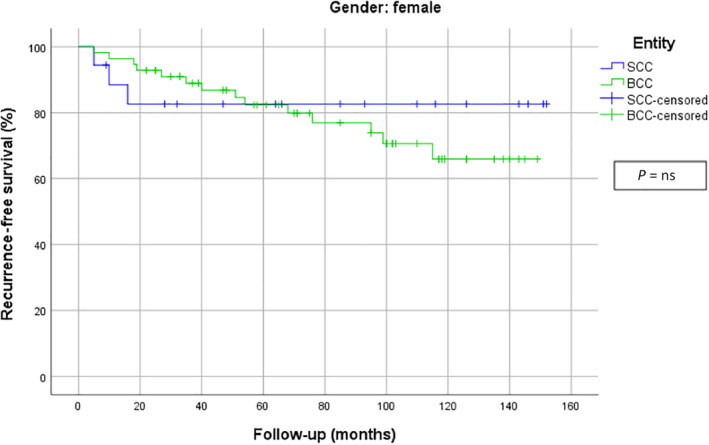
Recurrence‐free survival curves for women [Color figure can be viewed at wileyonlinelibrary.com]

When the whole study population was considered, the recurrence‐free survival curve showed earlier recurrences of cSCC in contrast to BCCs. Interestingly cSCCs, who at first showed numerous relapses, present a plateau phase after 2 years. However, BCC displayed a steady recurrence rate. After about 10 years, the recurrence‐free survival rate was nearly equal. Figure 4 shows the recurrence‐free survival curves for all patients with NMSC. When analyzing the recurrence‐free survival time exclusively for men, a significant difference could be detected: cSCC showed an earlier recurrence than BCCs (*P* = .027). Figure 5 shows the recurrence‐free survival curves of men. In contrast, the recurrence‐free survival curves for women showed most recurrences of cSCCs in the first 20 months, while BCC showed a more slowly but continuous rate of recurrence which finally crossed below the SCC curve. However, no statistically significant differences could be detected between these two groups. Figure 6 demonstrates the recurrence‐free survival curves of woman.

## 4 DISCUSSION

Our results demonstrate that for woman the distribution of BCC to cSCC is approximately 4:1, while in the male group the ratio of BCC to cSCC is approximately 1:1.1. In comparison to our findings, Ahmad et al, who included 41 patients with NMSC of the pinna in their study, presented an incidence of BCC to cSCC of 1.3:1.^32^ Tolkachjov who included 649 patients to their study described a ratio of BCC to cSCC of 1:1.3.^33^ Both studies did not execute any sex‐specific analysis. If the results of our study are considered exclusively for men, then they are consistent with these published findings. For us, it is of clinical importance to note that in female patients the chance of skin cancer on the pinna being a BCC is much higher than previous studies would suggest. To the best of our knowledge, we are the first to describe a significant difference of distribution of NMSC of the pinna between men and woman. These results can be helpful for the clinical diagnosis of NMSCs, in case of lesions difficult to confirm due to complex anatomic conditions.

Although cSCC and BCC share a lot of similarities, they have a difference in their etiological characteristic. A higher risk for BCC is suggested for frequent and severe sunburns in childhood and adolescent.^34^ Whereas the highest risk factor for cSCC is the cumulative dose of UV radiation.^35^ An attempt of explanation could be that women have longer hair which covers their ears and provides prevention against UV radiation. This might be a protective factor for cSCCs in women. But in cases of BCC, this possible explanation is not durable because women are concerned more often than men in our cohort. Adalsteinsson et al describe a significant higher incidence of BCC in women than in men recently in the population of Iceland. They state that the increase of BCC in women correlates with the increase of traveling and use of tanning beds.^36^

A recent Skh‐1 mice model study showed different reactions to UV radiation in reference to inflammation and consecutive different antioxidant activity levels in male and female mice; and therefore resulting higher number and severity of carcinoma in male mice.^37^ Further molecular pathologic pathway studies about the genesis of NMSC and the immunologic reaction to UV radiation especially on sex‐dependent differences are needed.^38^, ^39^, ^40^ Over the last few decades, the risk of developing cSCC and BCC has risen significantly in women.^41^, ^42^

In our study, population men are affected twice as frequent by NMSC at the pinna than women. In reports of the American Cancer Society, an equal sex distribution has been described, however not explicit for the external ear but for the whole body.^43^

The recurrence rate of BCC was determined at 18.1% in our study population, which is slightly higher to the described recurrence rates of up to 14% in literature.^44^, ^45^ Furthermore, also not in concordance with our data is the fact that in general the incidence rate of BCC is approximately 20% higher in men than in woman.^42^ The recurrence rate for cSCC at 25.5% in our study is in accordance with former published studies, which report rates from 17% to 37%.^46^, ^47^

Nevertheless, all cutaneous carcinomas of the pinna are reported to be in a hazardous location for neoplasia and are therefore labeled high‐risk carcinoma with an increased risk of recurrence and morbidity.^33^, ^47^ This knowledge from multiple earlier publications is consistent with the data of this study. Therefore, it is of crucial importance to prevent the occurrence of NMSC by reducing the risk through better public education on the dangers of UV radiation and the importance of effective protection.

## 5 CONCLUSION

In our study, a new aspect on the sex distribution of cSCC and BCC of the pinna could be demonstrated. The knowledge, that women are concerned four times as frequent by BCC than by cSCC and men are affected approximately equally often by the most common NMSCs, should be used carefully in risk evaluation of suspect lesions at the pinna.

## CONFLICT OF INTEREST

The authors declare no potential conflict of interest.

## References

[1] Alam M , Nanda S , Mittal BB , Kim NA , Yoo S . The use of brachytherapy in the treatment of nonmelanoma skin cancer: a review. J Am Acad Dermatol. 2011;65(2):377‐388. 21496952 10.1016/j.jaad.2010.03.027

[2] Samarasinghe V , Madan V . Nonmelanoma skin cancer. J Cutan Aesthet Surg. 2012;5(1):3‐10. 22557848 10.4103/0974-2077.94323 PMC3339125

[3] Rogers HW , Weinstock MA , Harris AR , et al. Incidence estimate of nonmelanoma skin cancer in the United States, 2006. Arch Dermatol. 2010;146(3):283‐287. 20231499 10.1001/archdermatol.2010.19

[4] Katalinic A , Kunze U , Schafer T . Epidemiology of cutaneous melanoma and non‐melanoma skin cancer in Schleswig‐Holstein, Germany: incidence, clinical subtypes, tumour stages and localization (epidemiology of skin cancer). Br J Dermatol. 2003;149(6):1200‐1206. 14674897 10.1111/j.1365-2133.2003.05554.x

[5] Garcia C , Poletti E , Crowson AN . Basosquamous carcinoma. J Am Acad Dermatol. 2009;60(1):137‐143. 19103364 10.1016/j.jaad.2008.09.036

[6] Villani A , Fabbrocini G , Costa C , Carmela Annunziata M , Scalvenzi M . Merkel cell carcinoma: therapeutic update and emerging therapies. Dermatol Ther. 2019;9:209‐222. 10.1007/s13555-019-0288-z PMC6522614 30820877

[7] McGuire JF , Ge NN , Dyson S . Nonmelanoma skin cancer of the head and neck I: histopathology and clinical behavior. Am J Otolaryngol. 2009;30(2):121‐133. 19239954 10.1016/j.amjoto.2008.03.002

[8] Gustaityte‐Larsen D , Illum P . Non‐melanoma skin cancer of the auricle is treated according to national guidelines. Dan Med J. 2013;60(3):A4587. 23484609

[9] Eisemann N , Waldmann A , Geller AC , et al. Non‐melanoma skin cancer incidence and impact of skin cancer screening on incidence. J Invest Dermatol. 2014;134(1):43‐50. 23877569 10.1038/jid.2013.304

[10] Wong CS , Strange RC , Lear JT . Basal cell carcinoma. BMJ. 2003;327(7418):794‐798. 14525881 10.1136/bmj.327.7418.794 PMC214105

[11] Stratigos A , Garbe C , Lebbe C , et al; European Dermatology Forum (EDF), European Association of Dermato‐Oncology (EADO), European Organization for Research and Treatment of Cancer (EORTC)Diagnosis and treatment of invasive squamous cell carcinoma of the skin: European consensus‐based interdisciplinary guideline. Eur J Cancer. 2015;51(14):1989‐2007. 26219687 10.1016/j.ejca.2015.06.110

[12] Alam M , Ratner D . Cutaneous squamous‐cell carcinoma. N Engl J Med. 2001;344(13):975‐983. 11274625 10.1056/NEJM200103293441306

[13] Miller DL , Weinstock MA . Nonmelanoma skin cancer in the United States: incidence. J Am Acad Dermatol. 1994;30(5 pt 1):774‐778. 8176018 10.1016/s0190-9622(08)81509-5

[14] Chen AC , Halliday GM , Damian DL . Non‐melanoma skin cancer: carcinogenesis and chemoprevention. Pathology. 2013;45(3):331‐341. 23478234 10.1097/PAT.0b013e32835f515c

[15] Kripke ML . Immunological effects of ultraviolet radiation. J Dermatol. 1991;18(8):429‐433. 1761789 10.1111/j.1346-8138.1991.tb03111.x

[16] Euvrard S , Kanitakis J , Claudy A . Skin cancers after organ transplantation. N Engl J Med. 2003;348(17):1681‐1691. 12711744 10.1056/NEJMra022137

[17] Lear JT , Tan BB , Smith AG , et al. Risk factors for basal cell carcinoma in the UK: case‐control study in 806 patients. J R Soc Med. 1997;90(7):371‐374. 9290417 10.1177/014107689709000704 PMC1296380

[18] Xie J , Murone M , Luoh SM , et al. Activating smoothened mutations in sporadic basal‐cell carcinoma. Nature. 1998;391(6662):90‐92. 9422511 10.1038/34201

[19] Box NF , Duffy DL , Irving RE , et al. Melanocortin‐1 receptor genotype is a risk factor for basal and squamous cell carcinoma. J Invest Dermatol. 2001;116(2):224‐229. 11179997 10.1046/j.1523-1747.2001.01224.x

[20] Baez CF , Goncalves MTV , da Rocha WM , et al. Investigation of three oncogenic epitheliotropic viruses shows human papillomavirus in association with non‐melanoma skin cancer. Eur J Clin Microbiol Infect Dis. 2019;38:1129‐1133. 30788731 10.1007/s10096-019-03508-z

[21] Criscione VD , Weinstock MA , Naylor MF , Luque C , Eide MJ , Bingham SF . Actinic keratoses: natural history and risk of malignant transformation in the Veterans Affairs Topical Tretinoin Chemoprevention Trial. Cancer. 2009;115(11):2523‐2530. 19382202 10.1002/cncr.24284

[22] Marks R , Rennie G , Selwood TS . Malignant transformation of solar keratoses to squamous cell carcinoma. Lancet. 1988;1(8589):795‐797. 2895318 10.1016/s0140-6736(88)91658-3

[23] Gloster HM Jr , Brodland DG . The epidemiology of skin cancer. Dermatol Surg. 1996;22(3):217‐226. 8599733 10.1111/j.1524-4725.1996.tb00312.x

[24] Fahradyan A , Howell AC , Wolfswinkel EM , Tsuha M , Sheth P , Wong AK . Updates on the management of non‐melanoma skin cancer (NMSC). Healthcare. 2017;5(4):82. 10.3390/healthcare5040082 PMC5746716 29104226

[25] Paolino G , Cardone M , Didona D , et al. Prognostic factors in head and neck melanoma according to facial aesthetic units. G Ital Dermatol Venereol. 2020;155(1):41‐45. 28748684 10.23736/S0392-0488.17.05685-1

[26] Ting PT , Kasper R , Arlette JP . Metastatic basal cell carcinoma: report of two cases and literature review. J Cutan Med Surg. 2005;9(1):10‐15. 16208438 10.1007/s10227-005-0027-1

[27] Clark RR , Soutar DS , Hunter KD . A retrospective analysis of histological prognostic factors for the development of lymph node metastases from auricular squamous cell carcinoma. Histopathology. 2010;57(1):138‐146. 20653785 10.1111/j.1365-2559.2010.03593.x

[28] Yoon M , Chougule P , Dufresne R , Wanebo HJ . Localized carcinoma of the external ear is an unrecognized aggressive disease with a high propensity for local regional recurrence. Am J Surg. 1992;164(6):574‐577. 1463102 10.1016/s0002-9610(05)80709-3

[29] Lewis KG , Weinstock MA . Nonmelanoma skin cancer mortality (1988‐2000): the Rhode Island follow‐back study. Arch Dermatol. 2004;140(7):837‐842. 15262694 10.1001/archderm.140.7.837

[30] Harris BN , Bayoumi A , Rao S , Moore MG , Farwell DG , Bewley AF . Factors associated with recurrence and regional adenopathy for head and neck cutaneous squamous cell carcinoma. Otolaryngol Head Neck Surg. 2017;156(5):863‐869. 28322123 10.1177/0194599817697053

[31] Prieto‐Granada C , Rodriguez‐Waitkus P . Cutaneous squamous cell carcinoma and related entities: epidemiology, clinical and histological features, and basic science overview. Curr Probl Cancer. 2015;39(4):206‐215. 26239204 10.1016/j.currproblcancer.2015.07.005

[32] Ahmad I , Das Gupta AR . Epidemiology of basal cell carcinoma and squamous cell carcinoma of the pinna. J Laryngol Otol. 2001;115(2):85‐86. 11320842 10.1258/0022215011907497

[33] Tolkachjov SN . Utilization of topographical Mohs micrographic surgery maps for rapid review of clinicopathologic characteristics of nonmelanoma skin cancers of the ear. Dermatol Surg. 2018;44(1):25‐30. 28858932 10.1097/DSS.0000000000001244

[34] Corona R , Dogliotti E , D'Errico M , et al. Risk factors for basal cell carcinoma in a Mediterranean population: role of recreational sun exposure early in life. Arch Dermatol. 2001;137(9):1162‐1168. 11559211 10.1001/archderm.137.9.1162

[35] de Vries E , Trakatelli M , Kalabalikis D , et al; on behalf of the EPIDERM GroupKnown and potential new risk factors for skin cancer in European populations: a multicentre case‐control study. Br J Dermatol. 2012;167(suppl 2):1‐13. 10.1111/j.1365-2133.2012.11081.x 22881582

[36] Adalsteinsson JA , Ratner D , Olafsdottir E , et al. Basal cell carcinoma: an emerging epidemic in women in Iceland. Br J Dermatol. 2020 10.1111/bjd.18937. 32030719

[37] Thomas‐Ahner JM , Wulff BC , Tober KL , Kusewitt DF , Riggenbach JA , Oberyszyn TM . Gender differences in UVB‐induced skin carcinogenesis, inflammation, and DNA damage. Cancer Res. 2007;67(7):3468‐3474. 17389759 10.1158/0008-5472.CAN-06-3798

[38] Alameda JP , Gaspar M , Ramirez A , et al. Deciphering the role of nuclear and cytoplasmic IKKα in skin cancer. Oncotarget. 2016;7(20):29531‐29547. 27121058 10.18632/oncotarget.8792 PMC5045415

[39] Farzan SF , Karagas MR , Christensen BC , Li Z , Kuriger JK , Nelson HH . RNASEL and MIR146A SNP‐SNP interaction as a susceptibility factor for non‐melanoma skin cancer. PLoS One. 2014;9(4):e93602. 24699816 10.1371/journal.pone.0093602 PMC3974770

[40] Feehan RP , Nelson AM , Shantz LM . Inhibition of mTORC2 enhances UVB‐induced apoptosis in keratinocytes through a mechanism dependent on the FOXO3a transcriptional target NOXA but independent of TRAIL. Cell Signal. 2018;52:35‐47. 30172026 10.1016/j.cellsig.2018.08.018 PMC6185741

[41] Christensen GB , Ingvar C , Hartman LW , Olsson H , Nielsen K . Sunbed use increases cutaneous squamous cell carcinoma risk in women: a large‐scale, prospective study in Sweden. Acta Derm Venereol. 2019;99(10):878‐883. 31017252 10.2340/00015555-3198

[42] Muzic JG , Schmitt AR , Wright AC , et al. Incidence and trends of basal cell carcinoma and cutaneous squamous cell carcinoma: a population‐based study in Olmsted County, Minnesota, 2000 to 2010. Mayo Clin Proc. 2017;92(6):890‐898. 28522111 10.1016/j.mayocp.2017.02.015 PMC5535132

[43] Oberyszyn TM . Non‐melanoma skin cancer: importance of gender, immunosuppressive status and vitamin D. Cancer Lett. 2008;261(2):127‐136. 18267352 10.1016/j.canlet.2008.01.009

[44] Kumar P , Orton CI , McWilliam LJ , Watson S . Incidence of incomplete excision in surgically treated basal cell carcinoma: a retrospective clinical audit. Br J Plast Surg. 2000;53(7):563‐566. 11000071 10.1054/bjps.2000.3394

[45] Rieger KE , Linos E , Egbert BM , Swetter SM . Recurrence rates associated with incompletely excised low‐risk nonmelanoma skin cancer. J Cutan Pathol. 2010;37(1):59‐67. 19615009 10.1111/j.1600-0560.2009.01340.x

[46] Karia PS , Morgan FC , Ruiz ES , Schmults CD . Clinical and incidental perineural invasion of cutaneous squamous cell carcinoma: a systematic review and pooled analysis of outcomes data. JAMA Dermatol. 2017;153(8):781‐788. 28678985 10.1001/jamadermatol.2017.1680 PMC5657475

[47] Burton KA , Ashack KA , Khachemoune A . Cutaneous squamous cell carcinoma: a review of high‐risk and metastatic disease. Am J Clin Dermatol. 2016;17(5):491‐508. 27358187 10.1007/s40257-016-0207-3

